# Interferon add-on therapy increased clinical cure significantly for interferon-experienced chronic hepatitis B patients with low HBsAg

**DOI:** 10.3389/fimmu.2022.997608

**Published:** 2022-09-06

**Authors:** Xiaoan Yang, Ka Zhang, Qihuan Xu, Xin Shu, Zhishuo Mo, Dongying Xie, Zhiliang Gao, Hong Deng

**Affiliations:** ^1^ Department of Infectious Diseases, The Third Affiliated Hospital of Sun Yat-Sen University, Guangzhou, China; ^2^ Guangdong Provincial Key Laboratory of Liver Disease Research, The Third Affiliated Hospital of Sun Yat-sen University, Guangzhou, China

**Keywords:** interferon add-on therapy, clinical cure, interferon-experienced patients, chronic hepatitis B patients (CHB), HBsAg

## Abstract

Currently, interferon add-on therapy brings hope for clinical cure of chronic hepatitis B patients with low HBsAg. However, in clinical practice patients with poor responses to their first interferon therapy were often switched to nucleos(t)ide analog therapy and then labeled as unsuitable patients for interferon therapy. Even if their HBsAg levels dropped to a low level, they were reluctant or not recommended to take interferon again, which caused them to miss out on interferon add-on therapy and clinical cure. Therefore, it is urgent to elucidate the effectiveness of interferon add-on therapy to get clinical cure for these interferon-experienced patients with low HBsAg. The purpose of this study was to investigate whether interferon-experienced patients could achieve the same HBsAg clearance and HBsAg seroconversion rates as interferon-naive patients. Also, the associated factor of HBsAg clearance and seroconversion were aimed to be clarified. 292 patients, including 85 interferon-experienced patients, were enrolled with HBsAg< 1500 IU/ml, HBeAg negative and HBV-DNA negative. And then, peg-interferon α-2b add-on therapy was performed. The results showed that the week 48 HBsAg clearance and seroconversion rates of all patients were 29.8% and 22.0%. There was no statistically significant difference between interferon-experienced and interferon-naive patients in week 48 HBsAg clearance and seroconversion rates, suggesting satisfactory clinical cure of the interferon add-on therapy for interferon-experienced patients. The age, baseline HBsAg, and week 12 HBsAg were negative correlated factors for week 48 HBsAg clearance and seroconversion. Furthermore, the age, baseline HBsAg and week 12 HBsAg for predicting the week 48 HBsAg clearance were cut off at 40.5 years, at 152.0 IU/ml and at 34.99 IU/ml, and for predicting seroconversion were cut off at 40.5 years, at 181.9 IU/ml and at 34.99 IU/ml, correspondingly. Significantly, interferon-experienced patients with low HBsAg were suggested with interferon add-on therapy to achieve clinical cure as soon as possible. This research provided evidences and cut-offs for the interferon add-on therapy against chronic hepatitis B.

## Introduction

Chronic hepatitis B virus (HBV) infection leads to hepatitis B virus related end-stage liver disease and then death (1, 2), causing great burdens for patients and their economics. HBsAg clearance, with or without seroconversion, is the criteria of clinical cure for patients with chronic HBV infection and then associated with improved long-term prognosis in chronic hepatitis B (3). Although with negative HBeAg and low HBV-DNA levels, patients with higher HBsAg levels (≥1000 IU/ml) are still at a higher risk of developing into hepatocellular carcinoma (4, 5). Even for patients with HBsAg<100 IU/ml, their virological recurrence rate within 1 year after discontinuation of nucleos(t)ide analogs therapies is still as high as 9.1%-19.6% (6). Compared with patients with persistent positive HBsAg, patients with HBsAg clearance are less likely to develop cirrhosis and liver cancer (7). Therefore, clinical cure (HBsAg clearance with or without seroconversion) is regarded as the ideal treatment goal of chronic hepatitis B treatment in many crucial guidelines (8–10) instead of low HBsAg levels.

Clinically, first-line antiviral drugs include immunomodulators (peg-interferon) and drugs directly act on different targets in the viral replication cycle, taken as nucleos(t)ide analogs as examples. Currently, the first-line nucleos(t)ide analogs, such as ETV, TDF, TAF, can effectively inhibit HBV replication; however, they are difficult to inhibit the transcriptional activity of covalently closed circular DNA (cccDNA) (11, 12). Moreover, the clearance rates of HBsAg in nucleos(t)ide analogs therapies are as low as 0-3% (13), and most cannot achieve clinical cure. The other therapy, interferon, has dual effects of direct antiviral and immune regulation; however, interferon alone is only effective for some patients, and the HBsAg clearance rate of peg-IFN monotherapy is only 3%-7% (14). Thus, either nucleos(t)ide analogs monotherapy or interferon monotherapy is not suitable.

Immunologically, the combination of interferon and nucleos(t)ide analogs is based on a rigid theoretical basis. Firstly, interferon mainly activates the innate immune response, enhancing the antiviral activity of natural killer NK cells. But it may lead to the depletion of effector CD8+ T cell function, limiting the recovery effect on HBV-specific CD8+ T cell function (15, 16). In addition, nucleos(t)ide analogs inhibited HBV replication and could directly enhance the effect of interferon-induced innate immune activation (17, 18). Thus, the combination of the two promotes functional recovery of HBV-specific T cells and innate and adaptive immune cells (19, 20). Hence, the reasonable combination of the two may play an additive or synergistic effect, which is highly wanted to be clarified.

Currently, many studies have been carried out on the rational combination of nucleos(t)ide analogs and interferon therapy to achieve clinical cure (21–25). For patients who were taking nucleos(t)ide analogs with negative HBV-DNA, negative HBeAg, and low HBsAg, the addition of interferon has been proved to be an effective and rational combination strategy, which can achieve higher clinical cure rate than nucleos(t)ide analogs or interferon alone. Our study also found that for chronic hepatitis B patients with negative HBeAg, negative HBV-DNA, and HBsAg< 1500 IU/ml, after receiving interferon add on therapy, the 48-week HBsAg clearance rate was as high as 36.7% (26). However, efficacy of interferon add-on therapy for interferon-experienced patients, who responded poorly to interferon and then switched to nucleos(t)ide analogs were unknown. Unfortunately, they refused or were advised against interferon add-on therapy by their clinicians because they had a poor response to first-time interferon therapy. Therefore, it is highly wanted to elucidate if they are appropriate for interferon add-on therapy to get clinical cure. On other hand, high HBsAg clearance rate can be achieved with interferon add-on therapy; however, it is unclear whether HBsAg seroconversion rate is satisfactory with interferon add-on therapy. Moreover, simple, clear, and clinically-operated indicators to assess whether patients are suitable for initiation or continuation of interferon-add on therapy are deficient.

In this study, hepatitis B patients with low HBsAg, including interferon-experienced patients, were enrolled to observe the efficacy and safety of the interferon add-on therapy, and also to screen the dominant population who would be more likely to achieve clinical cure. The suitability of interferon add-on therapy for interferon-experienced patients with low HBsAg was also explored. Furthermore, the predictive factors for clinical cure were investigated by logistic regression analysis and ROC curve plotting. This research may provide evidences and predictive factors for the interferon add-on therapies against chronic hepatitis B.

## Methods

### Patients

**Figure 1:**
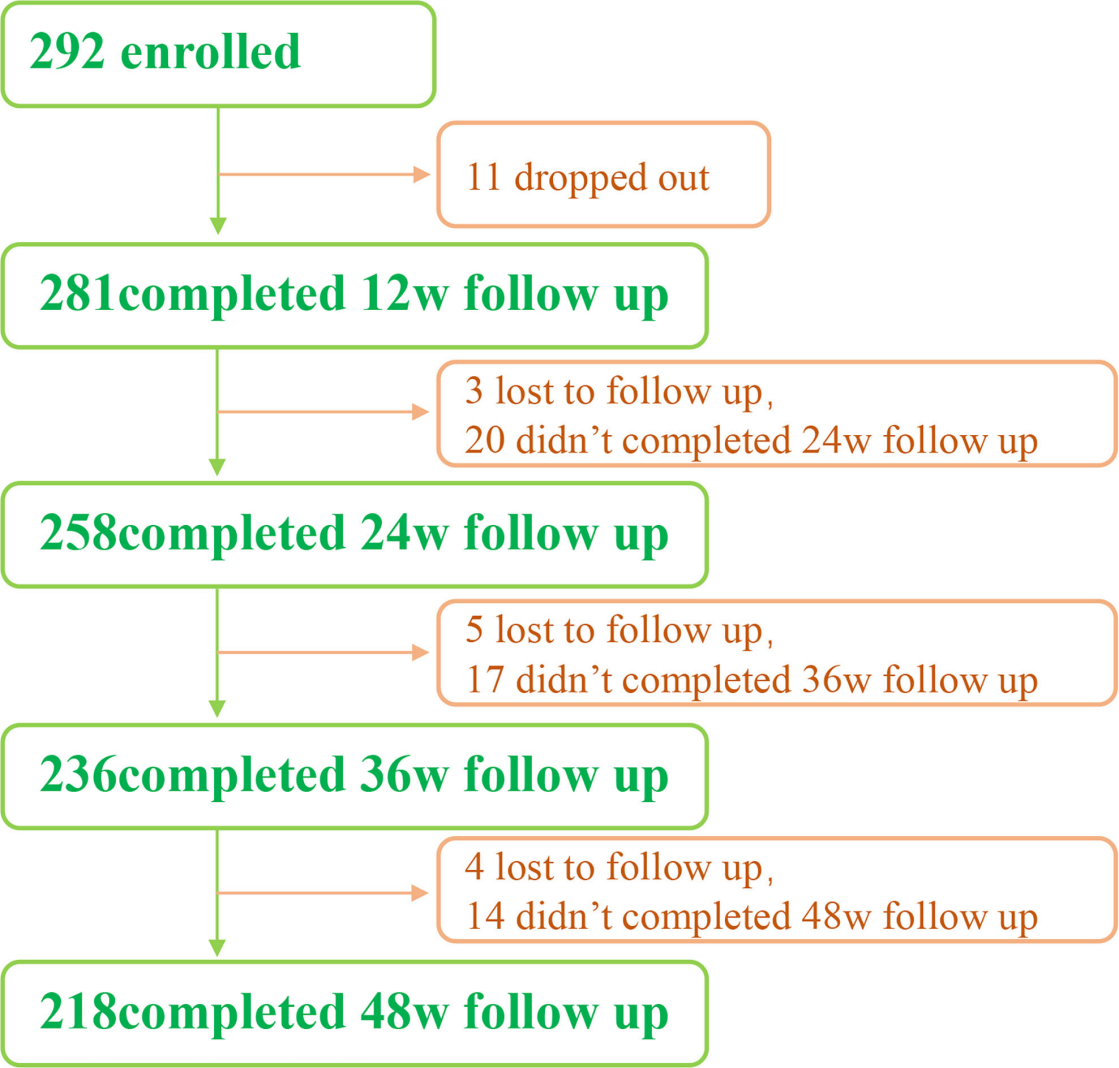
Flow chart of patients enrolled in this study.

From May 2018 to August 2021, we enrolled a total of 292 patients. All of them were from the outpatients of Infectious Diseases Department of the Third Affiliated Hospital of Sun Yat-Sen University. 218 people completed the 48-week follow-up, 11 dropped out, and 12 were lost to follow-up (**Figure 1**).

### Inclusion criteria

The inclusion criteria was set as the following: (1) age was set between 18-60 years old; (2) patients were previously diagnosed with hepatitis B virus infection and treated with nucleos(t)ide analogs therapy for more than 1 year, and two HBsAg tests were positive (> 0.05 IU/ml); (3) HBsAg< 1500 IU/ml, HBeAg negative (< 1 COI), HBeAb negative or positive, and HBV-DNA negative (< 20 copies/ml).

### Exclusion criteria

The exclusion criteria was set as the following: (1) baseline ALT > 10 ULN or TB > 2 ULN; (2) neutrophil count< 1.5 × 10e9/L or platelet count< 70 × 10e9/L; (3) allergic to interferon-α2b or its components; (4) hepatitis B-related decompensated cirrhosis or liver cancer; (5) combined with hepatitis C, hepatitis E, alcoholic liver disease, autoimmune liver disease, PBC and other diseases causes liver damage; (6) pregnancy or plan to be pregnant in the short term; (7) history of mental illness; (8) retinal disease; (9) autoimmune disease; (10) thyroid disease; (11) uncontrolled underlying diseases such as diabetes, hypertension, or severe heart, lung or kidney diseases.

### Exit criteria

The patients with the following circumstances should be exited: (1) serious adverse events or allergic reactions that may be related to the drug; (2) neutrophil count< × 10e9/L during medication or no improvement after interferon dose modification or suspension; (3) platelet< 25 × 10e9/L during medication or no improvement after interferon dose modification or suspension; (4) various mental symptoms; (5) The subject’s pregnancy test was positive; (6) the subject was lost to follow-up.

### Scheme

The patients (292 patients) enrolled were divided into two groups, interferon-experienced patients and interferon-naive patients (**Table 1**). Interferon-experienced patients (85 patients) meant patients who used peg-interferon therapies for more than 24 weeks but did not achieve clinical cure or HBV-DNA clearance, and switched to nucleos(t)ide analogs for more than 1 year before enrollment, and then met the inclusion criteria of this study. Interferon-naive patients (207 patients) meant patients who never used interferon but used nucleos(t)ide analogs for more than 1 year before enrollment, and then met the inclusion criteria of this study. Upon enrollment, all patients were added interferon on the basis of the original medication, which was defined as interferon add-on therapy.

**Table 1:** Baseline characteristics.

	All patientsn=292	interferon-experienced n=85	interferon-naive n=207	P*
sex (male)	263 (90.1%)	77 (90.6%)	186 (89.9%)	1.000
Age	44 (38-49)	41 (36-45)	45 (39-50)	0.000*
DNA-negative duration	41.5 (18-70.5)	31 (18-51)	45 (20-81)	0.014*
ALT	25 (20-34)	23 (19-31)	27 (21-35.5)	0.056
AST	24 (20-28)	21 (19-26)	24 (21-29)	0.003*
TB	10.8 (7.8-13.8)	10.3 (7.6-13.4)	10.9 (7.85-13.8)	0.509
ALB	48.54 ± 2.61	48.57 ± 2.55	48.52 ± 2.64	0.888
WBC	6.03 (5.14-6.91)	5.78 (5.07-6.75)	6.13 (5.24-6.96)	0.090
HGB	156 (147-163)	156 (147-163.5)	156 (147-163)	0.526
PLT	208 (173-247)	218 (182-253)	205 (171.5-245)	0.162
HBeAb (+)	253 (86.6%)	71 (83.5%)	182 (87.9%)	0.345
HBsAg	268.1 (80.77-682.16)	212 (52-620)	273 (96.2-691.85)	0.193
Interferon combination upon enrollment				
Combined ETV	99 (33.9%)	22 (25.9%)	77 (37.2%)	0.077
Combined TDF	155 (53.1%)	48 (56.5%)	107 (51.7%)	0.519
Combined TAF	32 (11.0%)	12 (14.1%)	20 (9.7%)	0.303
Interferon alone	6 (2.1%)	3 (3.5%)	3 (1.4%)	0.362

Most data were expressed as n (%) or median (interquartile range), ALB is expressed as mean ± standard deviation. No statistical differences in baseline characteristics between the two groups except age, DNA-negative duration and AST. *P< 0.05 was considered statistically significant. DNA, hepatitis B virus DNA; ALT, alanine aminotransferase; AST, alanine aminotransferase; ALB, albumin; WBC, white blood cell; HGB, hemoglobin; PLT, platelets; HBeAb, hepatitis B e antibody; HBsAg, hepatitis B surface antigen; ETV, entecavir; TDF, tenofovir disoproxil fumarate; TAF, tenofovir alafenamide fumarate.

For the interferon add-on therapy, all patients received peg-interferon-α2b add-on therapy for 48 weeks upon enrollment. In detail, patients received subcutaneous injection of peg-interferon-α2b once a week, each dose was 180 μg. For a small number of patients with body weight less than 50 kg, the dose was adjusted to 135μg each time. After one month of treatment without obvious side effect, the dose was changed to 180 μg each time.

Specifically, a total of 267 patients (76 interferon-experienced patients; 191 interferon-naive patients) who used first-line nucleos(t)ide analogs, such as ETV, TDF and TAF before enrollment, continued to use the previous nucleos(t)ide analogs in combination with interferon upon enrollment.

A total of 19 patients (6 interferon-experienced patients; 13 interferon-naive patients) who used second-line nucleos(t)ide analogs before enrollment, including LAM, ADV and LDT, were switched to ETV, TDF or TAF according to the actual clinical situation in combination with interferon upon enrollment.

To sum up, the number of patients (82 interferon-experienced patients; 204 interferon-naive patients) who received interferon add-on therapy combined with ETV, TDF, and TAF upon enrollment was 99, 155, and 32, respectively.

A total of 6 people, who did use nucleos(t)ide analogs before enrollment but were not using nucleos(t)ide analogs at the time of enrollment, were treated with interferon alone upon enrollment.

Among them, there were 3 people in the interferon-experienced group. They have used interferon for more than 24 weeks, with poor effect, and switched to nucleos(t)ide analogs for more than 1 year. After the disease was controlled, they stopped the drug on their own or under the guidance of their doctors. At the time of enrollment, they were not taking any nucleos(t)ide analogs. And there were 3 people in the interferon-naive group. They took nucleos(t)ide analogs for more than 1 year before enrollment. After the disease was controlled, they stopped the drug on their own or under the guidance of their doctor. At the time of enrollment, they were not taking any nucleos(t)ide analogs either.

Laboratory tests were arranged at week 2, 4, 8, 12, 24, 36, and 48 to evaluate the efficacy and safety of the peg-interferon-α2b add-on therapies. In the event of side effects or abnormal laboratory tests, professional follow-up doctors would decide whether to give symptomatic treatment, adjust the dose of interferon, suspend or even stop the peg-interferon-α2b add-on therapy.

The clinical definition was set as the following: (1) clinical cure meant HBsAg clearance or seroconversion; (2) HBsAg clearance meant that HBsAg< 0.05 IU/ml and HBV-DNA negative; (3) HBsAg seroconversion meant HBsAg< 0.05 IU/ml, HBV-DNA negative, and HBsAb > 10 IU/L; (4) HBeAg seroconversion meant HBeAg negative and HBeAb positive;

### Statistical analysis

The data were analyzed by SPSS version 24.0 for Windows (SPSS Inc., Chicago, IL, USA). Normally distributed continuous variables were expressed as means ± standard error. Abnormally distributed continuous variables were expressed as median (interquartile range). And categorical variables were expressed as number (percentage). Mean of two continuous normally distributed variables were compared by independent samples Student’s test. Mean of two continuous abnormally distributed variables were compared by Mann-Whitney U nonparametric test. Proportions of two nominal variables were compared by Pearson’s χ2 test and Fisher’s exact test.

Logistic regression analysis was performed to analyze factors associated with HBsAg clearance and seroconversion. Based on the screened predictors, an equation was developed to predict the probability of HBsAg clearance and seroconversion in patients with low HBsAg treated with interferon. The ROC curve was generated to analyze the predictive probability, and the area under the ROC curve (AUC) was calculated.

## Results

### Baseline characteristics

The baseline characteristics were listed in **Table 1**. In detail, most of the patients enrolled were male (90.1%). Also, the median age was 44 years (IQR 38-49) and the majority of patients had achieved HBeAg seroconversion (86.6%). Moreover, the majority of patients were treated by peg-interferon α-2b combined with nucleos(t)ide analogs upon enrollment (97.9%).

### Patients follow-up

292 patients were included in the study (**Figure 1**). In which, 11 were dropped out and 12 were lost to follow-up. Finally, 218 patients completed the 48-week follow-up.

### Safety

The most common side effects in patients were increased ALT/AST (81.8%), leukopenia (41.1%), fever (39.0%) and neutropenia (36.7%). The incidence of most adverse effects was not significantly different between interferon-experienced and interferon-naive patients. Subjective symptoms such as fatigue and headache were less frequent in interferon-experienced patients, which may be related to the increased tolerance to interferon. 8.9% of patients modified the interferon dose or suspended interferon due to intolerable side effects, and 2.1% of patients discontinued the drug due to intolerable side effects. There were no clinical deaths (**Table 2**). The changes of liver function and blood routine indexes of patients at 12, 24, 36, and 48 weeks of treatment were shown in **Table 3**.

**Table 2:** Safety of the interferon add-on therapy.

	All patients n=292	Interferon-experienced n=85	interferon-naive n=207	*p*
leukopenia	120 (41.1)	30 (35.3)	90 (43.5)	0.197
Neutropenia	107 (36.7)	34 (40.0)	73 (35.3)	0.446
Thrombocytopenia	86 (29.5)	28 (32.9)	58 (28.0)	0.402
ALT/AST elevation	239 (81.8)	67 (78.8)	172 (83.1)	0.390
Fever	114 (39.0)	30 (35.3)	84 (40.6)	0.400
Fatigue	75 (25.7)	14 (16.5)	61 (29.2)	0.021*
Hair lost	47 (16.1)	9 (10.6)	38 (18.4)	0.101
Headache	36 (12.3)	6 (7.1)	30 (14.5)	0.079*
Influenza-like symptoms	30 (10.1)	6 (7.1)	24 (11.6)	0.246
Psychiatric symptoms	15 (5.1)	3 (3.5)	12 (5.8)	0.613
Interferon dose modification/suspension	26 (8.9)	6 (7.1)	20 (9.7)	0.478
Interferon discontinuation	6 (2.1)	1 (1.2)	5 (2.4)	0.475
death	0 (0)	0 (0)	0 (0)	

All data are expressed as n (%). *P< 0.05 was considered statistically significant. Leukopenia, WBC< 4.0×10e9/L; Neutropenia, Neutrophil count< 1.5×10e9/L; Thrombocytopenia, platelet count< 100×10e9/L; ALT/AST elevation, ALT/AST>80U/L; Influenza-like symptoms, nasal congestion, runny nose, muscle aches, chills, dizziness, etc. Psychiatric symptoms, apathy, anxiety, depression, hallucinations, etc. Interferon dose modification/suspension, when intolerable side effects occurred, the dose of interferon was reduced to half of the original dose, that was, 90μg each time, or the use of interferon was suspended for 1-2 weeks.

**Table 3:** Changes of liver function and blood routine indexes.

	week 0	week 12	week 24	week 36	week 48
ALT	25 (20-34)	57 (38-96)	49 (34-81.5)	42 (29-66)	38 (26.5-60.5)
AST	24 (20-28)	48 (35-74)	46 (34-64.5)	39 (30-60)	37 (28-53)
WBC	6.03 (5.14-6.91)	2.99 (2.51-3.65)	3.16 (2.51-4.03)	3.06 (2.52-4.01)	3.14 (2.57-4.11)
HGB	156 (147-163)	138 (128-146)	135 (125-144)	133 (124-142)	133 (122-143.5)
PLT	208 (173-247)	113 (85-138)	114 (88-140)	117 (87-146)	124 (97-154.5)

All data are expressed as median (interquartile range), ALT, alanine aminotransferase; AST, alanine aminotransferase; WBC, white blood cell; HGB, hemoglobin; PLT, platelets.

### HBsAg clearance rates

**Figure 2:**
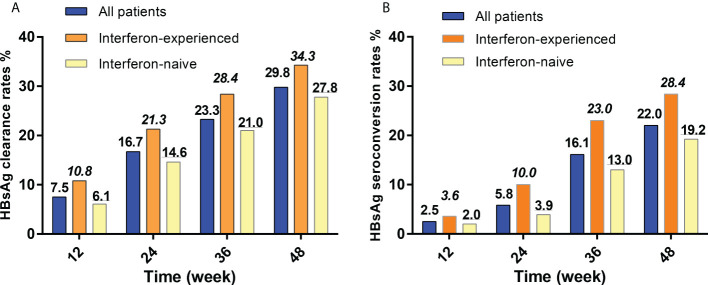
HBsAg clearance and seroconversion rates of interferon-experienced and interferon-naive patients. **(A)** HBsAg clearance rates of interferon-experienced and interferon-naive patients. **(B)** HBsAg seroconversion rates of interferon-experienced and interferon-naive patients. HBsAg, hepatitis B surface antigen. Interferon-naive patients meant patients who never used interferon but used nucleos(t)ide analogs therapies for more than 1 year and then met the inclusion criteria of this study; Interferon-experienced patients meant patients who used peg-interferon therapies for more than 24 weeks, did not achieve clinical cure or HBV-DNA clearance, and switched to nucleos(t)ide analogs for more than 1 year, and then met the inclusion criteria of this study.

At 12, 24, 36, and 48 weeks, the cumulative HBsAg clearance rates were 7.5%, 16.7%, 23.3% and 29.8% for all patients, 10.8%, 21.3%, 28.4% and 34.3% for interferon-experienced patients, and 6.1%, 14.6%, 21.0% and 27.8% for interferon-nave patients (**Figure 2A**). There was no statistical difference between the two groups at each time point in HBsAg clearance rates.

### HBsAg seroconversion rates

At 12, 24, 36, and 48 weeks, the cumulative HBsAg seroconversion rates were 2.5%, 5.8%, 16.1% and 22.0% for all patients, 3.6%, 10.0%, 23.0% and 28.4% for interferon-experienced patients, and 2.0%, 3.9%, 13.0% and 19.2% for interferon-naive patients (**Figure 2B**). There was no statistical difference between the two groups at each time point in HBsAg seroconversion rates.

### Logistic regression analysis of week 48 HBsAg clearance in all patients

To determine the relationship between baseline data and 48-week HBsAg clearance, logistic regression analysis was performed (**Table 4**). Baseline age and HBsAg were negative factors for week 48 HBsAg clearance, while gender, HBV-DNA negative duration, baseline ALT, AST, TB, ALB, HBeAg seroconversion, combined ETV or TDF or TAF, interferon alone, interferon-experienced were not associated factor for week 48 HBsAg clearance.

**Table 4:** Logistic regression analysis of week 48 HBsAg clearance in all patients.

Factors	B	S.E,	Wals	df	Sig	EXP(B)
age	-0.077	0.023	10.979	1	0.001	0.926
HBsAg	-0.004	0.001	30.113	1	0.000	0.996
constant	3.786	1.083	12.220	1	0.000	44.101

In this study, binary logistic regression was used to evaluate the effect of baseline characteristics on the week 48 HBsAg clearance of the study subjects. The obtained Logistic model was statistically significant with χ^2^ = 63.507 and P<0.001. The model was able to correctly classify 78.9% of the study subjects. Among the independent variables included in the model, age and HBsAg were statistically significant (P<0.05). Gender, HBV-DNA negative duration, ALT, HBeAb (+), combined ETV, combined TDF, combined TAF, and interferon-experienced were not statistically significant. The odds ratio of HBsAg clearance decreased by 7.4% for each one-year increase in age, and the odds ratio of HBsAg clearance decreased by 0.4% for each additional unit of HBsAg.

Based on the results of logistic regression, we constructed a risk score logit(P) that predicts whether HBsAg clearance could be achieved:


(1) Logit(P)=3.786−0.077*Age−0.004*HBsAg.

Then, it was calculated the probability of achieving week 48 HBsAg clearance for each subject based on logit(P):


(2) $$P=e^{\wedge }((3.786-0.077*Age-0.004*HBsAg))/[1+e^{\wedge }((3.786-0.077*Age-0.004*HBsAg))].$$

**Figure 3:**
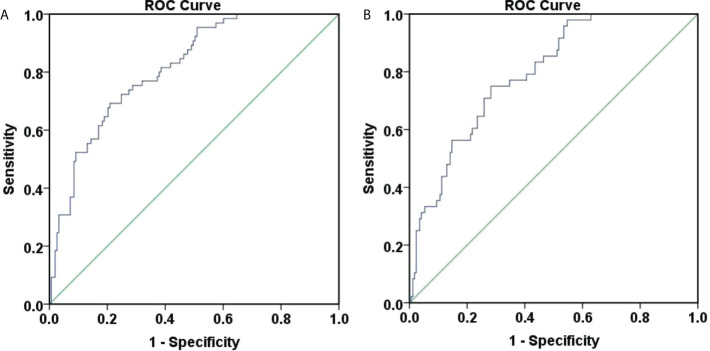
ROC curve for predicting 48-week HBsAg clearance and seroconversion. **(A)** ROC curve for predicting 48-week HBsAg clearance. The area under the curve was 0.814, 95% CI (0.756, 0.872), and the accuracy was good. The best cut-off value for the P value was 0.401, the Youden index was 0.483, the sensitivity was 0.692, and the specificity was 0.791; **(B)** ROC curve for predicting 48-week HBsAg seroconversion. The area under the curve was 0.794, 95% CI (0.729, 0.859), and the accuracy was good. The best cut-off value for the P value was 0.258, the Youden index was 0.468, the sensitivity was 0.750, and the specificity was 0.718.

Also, ROC curves were generated to assess the accuracy of P in predicting HBsAg clearance at 48 weeks (**Figure 3A**). The area under the curve was 0.814, 95% CI (0.756, 0.872), indicating a good accuracy. The best cut-off value for the P value was 0.401 and the corresponding Youden index, sensitivity and specificity were 0.483, 0.692, and 0.791. When the sensitivity was 95%, the P value was 0.598. And when the specificity was 95%, the P value was 0.103.

### Logistic regression analysis of week 48 HBsAg seroconversion in all patients

To determine the relationship between baseline data and 48-week HBsAg seroconversion, logistic regression analysis was performed. Baseline age, HBsAg were negative factors for week 48 HBsAg seroconversion, while gender, HBV-DNA negative duration, baseline ALT, AST, TB, ALB, HBeAg seroconversion, combined ETV or TDF or TAF, interferon alone, interferon-experienced were not an associated factor for week 48 HBsAg seroconversion (**Table 5**).

**Table 5:** Logistic regression analysis of week 48 HBsAg seroconversion in all patients.

Factors	B	S.E,	Wals	df	Sig	EXP(B)
age	-0.079	0.024	10.705	1	0.001	0.924
HBsAg	-0.004	0.001	21.759	1	0.000	0.996
constant	3.276	1.097	8.914	1	0.003	26.459

In this study, binary logistic regression was used to evaluate the effect of baseline characteristics on the week 48 HBsAg seroconversion of study subjects. The obtained Logistic model was statistically significant, χ2 = 46.999, P<0.001. The model was able to correctly classify 81.2% of the study subjects. Among the independent variables included in the model, age and HBsAg were statistically significant (P<0.05), gender, HBV-DNA negative duration, ALT, HBeAb(+), combined ETV, combined TDF, combined TAF, and interferon-experienced were not statistically significant. The odds ratio of HBsAg seroconversion decreased by 7.6% for each one-year increase in age, and the odds ratio of HBsAg seroconversion decreased by 0.4% for each additional unit of HBsAg.

Based on the results of logistic regression, we constructed a risk score logit(P) that predicts whether HBsAg seroconversion could be achieved:


(3) Logit(P)=3.276−0.079*Age−0.004*HBsAg.

Then, it was calculated the probability of achieving week 48 HBsAg seroconversion for each subject based on logit(P):


(4) $$P=e^{\wedge }((3.276-0.079*Age-0.004*HBsAg))/[1+e^{\wedge }((3.276-0.079*Age-0.004*HBsAg))].$$

Furthermore, ROC curves were generated to assess the accuracy of P in predicting HBsAg seroconversion at 48 weeks (**Figure 3B**). The area under the curve was 0.794, 95% CI (0.729, 0.859), suggesting a good accuracy. The best cut-off value for the P value was 0.258, corresponding Youden index, sensitivity and specificity at 0.468, 0.750, and 0.718. When the sensitivity was set at 95%, the P value was 0.499. When the specificity was 95%, the P value was 0.108.

### Predict week 48 HBsAg clearance and seroconversion with baseline age

**Figure 4:**
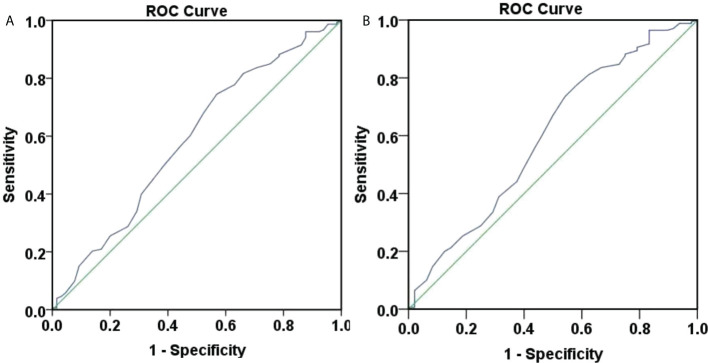
ROC curve of 48-weeks HBsAg clearance and seroconversion predicted by baseline age. **(A)** ROC curve of 48-weeks HBsAg clearance predicted by baseline age, the area under the curve was 0.586, 95% CI (0.500, 0.671), with poor accuracy. The optimal cut-off value for age was 40.5 years, the Youden index was 0.176, the sensitivity was 0.745, and the specificity was 0.431. When the sensitivity was 95%, the age was 30.5 years, and when the specificity was 95%, the age was 54.5 years. **(B)** ROC curve of 48-weeks HBsAg seroconversion predicted by baseline age, the area under the curve was 0.597, 95% CI (0.501, 0.692), with poor accuracy. The optimal cut-off value for age was 40.5 years, the Youden index was 0.193, the sensitivity was 0.735, and the specificity was 0.458. When the sensitivity was 95%, the age was 30.5 years, and when the specificity was 95%, the age was 53.5 years.

Although the above models presented good accuracies, it was difficult to implement in daily clinical work due to their complex calculation. Therefore, we assessed the effectiveness of baseline age in predicting week 48 HBsAg clearance and seroconversion with ROC curves, as shown in **Figure 4**.

**Figure 4A** showed that the area under the curve for baseline age to predict week 48 HBsAg clearance was 0.586, 95% CI (0.500, 0.671), suggesting a poor accuracy. The optimal cut-off value for age was 40.5 years, corresponding Youden index, sensitivity and specificity at 0.176, 0.745, and 0.431. When the sensitivity was 95%, the age was 30.5 years. When the specificity was 95%, the age was 54.5 years.

**Figure 4B** showed that the area under the curve for baseline age to predict week 48 HBsAg seroconversion was 0.597, 95% CI (0.501, 0.692), demonstrating a poor accuracy. The optimal cut-off value for age was 40.5 years and the corresponding Youden index, sensitivity and specificity, 0.193, 0.735, and 0.458. When the sensitivity was 95%, the age was 30.5 years. When the specificity was 95%, the age was 53.5 years.

### Predict week 48 HBsAg clearance and seroconversion with baseline HBsAg

**Figure 5:**
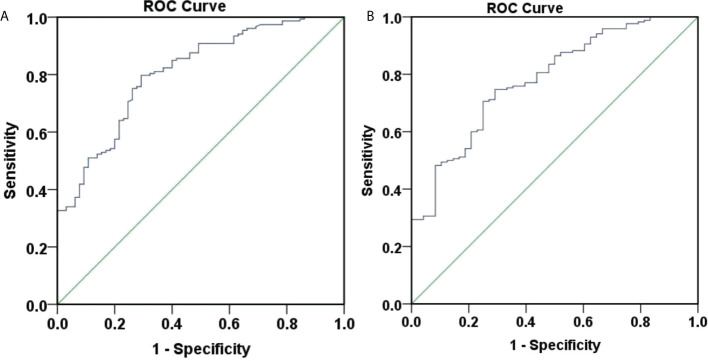
ROC curve of 48-weeksHBsAg clearance and seroconversion predicted by baseline HBsAg. **(A)** ROC curve of 48-weeks HBsAg clearance predicted by baseline HBsAg, the area under the curve was 0.807, 95% CI (0.746, 0.868), with good accuracy. The optimal cut-off value for HBsAg was 152.0 IU/ml, the Youden index was 0.505, the sensitivity was 0.797, and the specificity was 0.708. When the sensitivity was 95%, the HBsAg was 30.5 IU/ml, and when the specificity was 95%, the HBsAg was 679.0 IU/ml. **(B)** ROC curve of 48-weeks HBsAg seroconversion predicted by baseline HBsAg, the area under the curve was 0.782, 95% CI (0.711, 0.852), with average accuracy. The optimal cut-off value for HBsAg was 181.9 IU/ml, the Youden index was 0.456, the sensitivity was 0.706, and the specificity was 0.750. When the sensitivity was 95%, the HBsAg was 20.41 IU/ml, and when the specificity was 95%, the HBsAg was 711.85 IU/ml.

We also assessed the role of baseline HBsAg in predicting week 48 HBsAg clearance and seroconversion with ROC curves (**Figure 5**). **Figure 5A** showed that the area under the curve for baseline HBsAg to predict week 48 HBsAg clearance was 0.807, 95% CI (0.746, 0.868), showing a good accuracy. The optimal cut-off value for HBsAg was 152.0 IU/ml, corresponding Youden index, sensitivity and specificity to 0.505, 0.797, and 0.708. When the sensitivity was 95%, the HBsAg was 30.5 IU/ml. When the specificity was 95%, the HBsAg was 679.0 IU/ml. Overall, the baseline HBsAg showed a good predictive ability for week 48 HBsAg clearance.

**Figure 5B** showed that the area under the curve for baseline HBsAg to predict week 48 HBsAg seroconversion was 0.782, 95% CI (0.711, 0.852), depicting an average accuracy. The optimal cut-off value for HBsAg was 181.9 IU/ml and the corresponding Youden index, sensitivity and specificity were 0.456, 0.706, and 0.750. When the sensitivity was 95%, the HBsAg was 20.41 IU/ml. When the specificity was 95%, the HBsAg was 711.85 IU/ml.

### Predict week 48 HBsAg clearance with indicators at the early stage of treatment (week 12)

To determine the relationship between early stage (week 12) indicators and week 48 HBsAg clearance, logistics regression analysis was performed (**Table 6**). The results showed that Week 12 HBsAg was a negatively correlated factor for week 48 HBsAg clearance, while Week 12 HBsAg change from baseline, week 12 ALT, and Week 12 ALT elevation from baseline were not the associated factors for week 48 HBsAg clearance.

**Table 6:** Predict week 48 HBsAg clearance with week 12 indicators.

Factors	B	S.E,	Wals	df	Sig	EXP(B)
week 12 HBsAg	-0.012	0.002	24.615	1	0.000	0.988
constant	0.479	0.220	4.733	1	0.030	1.614

In this study, binary logistic regression was used to evaluate the effect of week 12 indicators on the week 48 HBsAg clearance of study subjects. The obtained Logistic model was statistically significant, χ^2^ = 84.432, P<0.001. The model was able to correctly classify 84.4% of the study subjects. Among the independent variables included in the model, week 12 HBsAg was statistically significant(P<0.05), Week 12 HBsAg change from baseline, week 12 ALT, and Week 12 ALT elevation from baseline were not statistically significant. The odds ratio of HBsAg clearance decreased by 1.2% for each additional unit of week 12 HBsAg.

**Figure 6:**
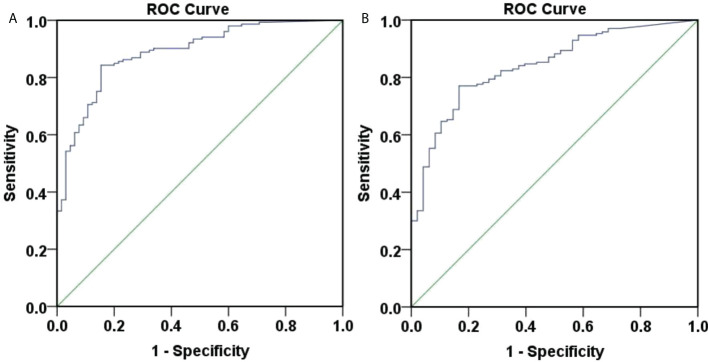
ROC curve of 48-weeks HBsAg clearance and seroconversion predicted by 12-week HBsAg. **(A)** ROC curve of 48-weeks HBsAg clearance predicted by 12-week HBsAg. The area under the curve was 0.888, 95% CI (0.843, 0.934), with good accuracy. The optimal cut-off value of week 12 HBsAg was 34.99 IU/ml, the Youden index was 0.689, the sensitivity was 0.843, and the specificity was 0.846. When the sensitivity was 95%, the HBsAg value was 4.715 IU/ml, and when the specificity was 95%, the HBsAg value was 198.6 IU/ml. **(B)** ROC curve of 48-weeks HBsAg seroconversion predicted by 12-week HBsAg. The area under the curve was 0.842, 95% CI (0.785,0.900), with good accuracy. The optimal cut-off value of week 12 HBsAg was 34.99 IU/ml, the Youden index was 0.603, the sensitivity was 0.771, and the specificity was 0.833. When the sensitivity was 95%, the HBsAg value was 1.425 IU/ml, and when the specificity was 95%, the HBsAg value was 161.95 IU/ml.

ROC curves were produced to assess the accuracy of week 12 HBsAg for predicting week 48 HBsAg clearance (**Figure 6A**). The area under the curve was 0.888, 95% CI (0.843, 0.934), depicting a good accuracy. The optimal cut-off value of week 12 HBsAg was 34.99 IU/ml, corresponding Youden index, sensitivity and specificity at 0.689, 0.843, and 0.846. When the sensitivity was set at 95%, the HBsAg value was at 4.715 IU/ml. Also, the HBsAg value was 198.6 IU/ml, when the specificity was 95%. These suggested a good accuracy of week 12 HBsAg for predicting week 48 HBsAg clearance.

### Predict week 48 HBsAg seroconversion with indicators at the early stage of treatment (week 12)

To determine the relationship between early stage (week 12) indicators and week 48 HBsAg seroconversion, logistics regression analysis was performed (**Table 7**). The results showed that week 12 HBsAg was a negative correlated factor for week 48 HBsAg seroconversion; while Week 12 HBsAg change, week 12 ALT, and Week 12 ALT elevation from baseline were not the associated factors for week 48 HBsAg seroconversion.

**Table 7:** Predict week 48 HBsAg seroconversion with week 12 indicators.

Factors	B	S.E,	Wals	df	Sig	EXP(B)
week 12 HBsAg	-0.010	0.002	16.775	1	0.000	0.990
constant	-0.196	0.218	0.810	1	0.368	0.822

In this study, binary logistic regression was used to evaluate the effect of week 12 indicators on the week 48 HBsAg seroconversion of study subjects. The obtained Logistic model was statistically significant, χ^2^ = 53.232, P< 0.001. The model was able to correctly classify 78.0% of the study subjects. Among the independent variables included in the model, week 12 HBsAg was statistically significant (P<0.05), Week 12 HBsAg change from baseline, week 12 ALT, and Week 12 ALT elevation from baseline were not statistically significant. The odds ratio of HBsAg seroconversion decreased by 1.0% for each additional unit of week 12 HBsAg.

ROC curves were plotted to assess the accuracy of week 12 HBsAg in predicting week 48 HBsAg seroconversion (**Figure 6B**). The area under the curve was 0.842, 95% CI (0.785, 0.900), depicting a good accuracy. The optimal cut-off values of week 12 HBsAg was 34.99 IU/ml, corresponding Youden index, sensitivity and specificity at 0.603, 0.771, and 0.833. Specifically, the HBsAg value was 1.425 IU/ml when the sensitivity was set at 95%. When the specificity was set at 95%, the HBsAg value was 161.95 IU/ml. These suggested a good accuracy of week 12 HBsAg in predicting week 48 HBsAg seroconversion.

## Discussion

Previously, it was reported that the interferon add-on therapies to chronic hepatitis B patients who had taken nucleos(t)ide analogs therapies with low HBsAg may lead to higher HBsAg clearance and seroconversion rates than interferon therapy or nucleos(t)ide analogs therapy alone (21–24). However, these studies were limited with small sized samples, varied inclusion criteria and treatment regiments, leading to fuzzy results. Therefore, we have carried out a more standardized, larger, prospective, real-world clinical study with the goal of finding the most suitable interferon therapy for the chronic hepatitis B population with low HBsAg. Our previous study found that interferon add-on therapy was more effective than interferon sequential therapy in low HBsAg chronic hepatitis B population (25), Hepatitis B patients with low HBsAg who receive interferon add-on therapy can obtain a relatively high HBsAg clearance rate (26).

In this paper, extended samples were enrolled and a standard criterion was used to analyze the effectiveness of the interferon add-on therapies on chronic hepatitis B patients with low HBsAg. It was worth to note that HBsAg clearance and seroconversion rates were reached up to 29.8% and 22.0% after 48 weeks peg-interferon α-2b add-on therapies for the patients of low-HBsAg by previous nucleos(t)ide analogs treatments. Not only the HBsAg clearance rate, but also the HBsAg seroconversion rate was quite satisfactory. HBsAb is a protective antibody, therefore, higher HBsAg seroconversion rate tends to mean less HBV reactivation and virological relapse. Evidently, interferon add-on therapy is beneficial to the clinical cure of chronic hepatitis B, which may be the joint effect of the inhibited virus replications by nucleos(t)ide analogs and the immune regulation by interferon (19, 20).

Specially, the safety and efficacy of interferon add-on therapy in interferon-experienced patients were the focus of this study. There was no difference in safety, HBsAg clearances and seroconversion rates of the interferon add-on therapies between the interferon-experienced patients and the interferon-naive patients. The results suggest that previous insensitivity to interferon is not a contraindication to interferon-add on therapy, nor does it suggest that secondary use of interferon is less effective. The situation before interferon treatment, such as HBsAg, HBeAg, HBV-DNA levels, etc., is the key to clinical cure. Also, resistance against interferon was not developed for first-time interferon therapies, which is well-known. Since the good efficacy, and acceptable safety, interferon-experienced patients with poor sensitivity to previous interferon treatment should not give up peg-interferon α2b add-on therapies to pursue clinical cure. Interferon-experienced patients should not be excluded when physicians screen suitable hepatitis B patients for interferon add-on therapy. Furthermore, it was suggested that the interferon treatment strategy against hepatitis B can be utilized from once in a lifetime to many tries.

Moreover, predictable factors for clinical cure are highly wanted for monitoring therapy processes. It was discovered that age and baseline HBsAg were negative correlated factors for clinical cure. Based on logistic regression analysis and ROC curve, a predictive model for HBsAg clearance and seroconversion was established for the peg-interferon α-2b add-on therapy in patients with low HBsAg. According to the patient’s age and baseline HBsAg, the probability of HBsAg clearance and seroconversion can be predicted, which is helpful for screening the dominant population who may benefit from the interferon add-on therapies. However, this model requires tedious calculations, so it is not applicable in clinical work practically. Therefore, we also assessed the possibility with only age or baseline HBsAg predicting treatment effect. HBsAg clearance and seroconversion rates of the interferon add-on therapies were less than 5% for patients older than 53.5 and 54.5 years, even if for patients with HBsAg of a low-level state. In contrast, the HBsAg clearance and seroconversion rates were greater than 95% for patients of low HBsAg and being younger than 30.5 years, similar to the previous study [24]. This may be caused by the decreases of immune cells with increasing age in terms of their number and function, which resulted in the weakening immune regulation function of interferon. Therefore, the interferon add-on therapies should be used as soon as possible to achieve clinical cure for suitable HBV-infected people. On the other hand, HBsAg clearance and seroconversion rates were less than 5% when the baseline HBsAg was greater than 679.0 and 711.85 IU/ml. However, HBsAg clearance and seroconversion rates were greater than 95% when baseline HBsAg was less than 30.5 and 20.41 IU/ml. These suggested that baseline HBsAg was closely related to the treatment efficacy, which was consistent to previous studies (22–24, 27). Therefore, interferon add-on therapy should be encouraged as soon as possible to achieve clinical cure for patients with low HBsAg.

Also, the effect of HBV-DNA negative duration was examined. It was found that the HBV-DNA negative duration was not an associated factor for HBsAg clearance or seroconversion for interferon add-on therapy. Therefore, if it was HBV-DNA negative, as long as HBsAg< 1500 IU/ml, the peg-interferon add-on therapy should be advised to pursue clinical cure and nucleos(t)ide analogs alone prolonged consolidation therapy was not required. Similarly, HBeAg seroconversion was not an associated factor for HBsAg clearance or seroconversion. If HBeAg turns negative, as long as HBsAg< 1500 IU/ml, the peg-interferon add-on therapy should be suggested. There’s no need to wait for HBeAg seroconversion to start peg-interferon add-on therapy. Also, the three currently used first-line nucleos(t)ide analogs (ETV, TDF, TAF) presented the same effect in the following peg-interferon add-on therapies. The type of nucleos(t)ide analogs did not play a big role, and there is no need to choose newer and more expensive drugs in order to pursue better therapeutic effects.

Furthermore, physicians need to predict the probability of clinical cure based on the patient’s in-treatment indicators and decide whether it is beneficial to continue the treatment after they start interferon add-on therapy. In this aspect, it was found that HBsAg at week 12 was associated closely with week 48 HBsAg clearance and seroconversion, which was similar to previous studies (24, 28, 29) and then may be used as the indicator for continuing or not continuing the interferon add-on therapies. When the week 12 HBsAg was greater than 198.6 or 161.95 IU/ml, the week 48 HBsAg clearance or seroconversion rate was less than 5%. For patients with poor response for the interferon add-on therapies, discontinuation of interferon should be considered.

## Conclusion

In this paper, we reported that patients with low HBsAg should be suggested for interferon add-on therapy to achieve clinical cure. Interferon-experienced patients who did not response well to the first interferon therapy also need to be advised for interferon add-on therapies. Age, baseline HBsAg and week 12 HBsAg were negatively correlated factors for week 48 HBsAg clearance and seroconversion, which were the dominant clinical cure indicators.

For the convenience for clinical reference, we roughly summarize the research results as the following: (1) if baseline age>55 years or baseline HBsAg>700 IU/ml, the probability of HBsAg clearance and seroconversion is extremely low and interferon add-on therapy is not recommended; (2) if baseline age<40 years or baseline HBsAg<180 IU/ml, the probability of HBsAg clearance and seroconversion is acceptable and interferon add-on therapy is recommended; (3) if baseline age<30 years or baseline HBsAg<30 IU/ml, the probability of HBsAg clearance and seroconversion is high and interferon add-on therapy is strongly recommended; (4) if week 12 HBsAg>200IU/ml, the probability of HBsAg clearance and seroconversion is extremely low and discontinuation of interferon should be suggested.

## Limitations

Our study has certain limitations, due to the high cost, only some patients were tested for HBV genotype. HBV genotype cannot be obtained due to low HBV-DNA load at enrollment. If the sample size of this study was larger and the follow-up period was longer, more complete data would be obtained. Due to the small number of interferon-experienced patients, the associated factors of clinical cure were not analyzed.

## Data availability statement

The original contributions presented in the study are included in the article/supplementary material. Further inquiries can be directed to the corresponding authors.

## Ethics statement

In 2018, we conducted a multi-center, open-label, real-world, observational, prospective, cohort study in China (http://www.chictr.org.cn, registration number: ChiCTR1800020369). All data in this article was from a subset of patients at one of the centers of the study. This study was approved by the Ethics Committee of the Third Affiliated Hospital of Sun Yat-Sen University, Guangzhou, China (the ethical number: [2018]02-218-01). The patients/participants provided their written informed consent to participate in this study.

## Author contributions

ZG and HD conceived and designed this study. ZG, HD, QX, and DX enrolled the patients. ZM, KZ, and XS followed up the patients. XY collected data and performed statistical analysis. XY and KZ drafted the manuscript. All authors contributed to the article and approved the submitted version.

## Funding

This work was supported by the Guangzhou Municipal Science and Technology Project, China (No. 202002030044 and 202103000060).

## Acknowledgments

Thanks to all the follow-up staff who participated in this study, without their hard work this study would not have been possible.

## Conflict of interest

The authors declare that the research was conducted in the absence of any commercial or financial relationships that could be construed as a potential conflict of interest.

## Publisher’s note

All claims expressed in this article are solely those of the authors and do not necessarily represent those of their affiliated organizations, or those of the publisher, the editors and the reviewers. Any product that may be evaluated in this article, or claim that may be made by its manufacturer, is not guaranteed or endorsed by the publisher.
